# Crystal structure of (*E*)-2-amino-4-methyl­sulfanyl-6-oxo-1-{[(thiophen-2-yl)­methyl­idene]­amino}-1,6-di­hydro­pyrimidine-5-carbo­nitrile

**DOI:** 10.1107/S205698901501885X

**Published:** 2015-10-14

**Authors:** Galal H. Elgemeie, Ali M. Salah, Reham A. Mohamed, Peter G. Jones

**Affiliations:** a Chemistry Department, Faculty of Science, Helwan University, Cairo, Egypt; b Institut für Anorganische und Analytische Chemie, Technische Universität Braunschweig, Postfach 3329, D-38023 Braunschweig, Germany

**Keywords:** crystal structure, pyrimidine, thien­yl, N—H⋯N and N—H⋯O hydrogen bonds.

## Abstract

The title compound, C_11_H_9_N_5_OS_2_, a 1-thio­phen-2-yl­methyl­ene­amino­pyrimidine derivative, displays an essentially planar C—NH_2_ group. The conformation across the N=C bond linking the pyrimidine and thienyl groups is *E*. The pyrimidine and thienyl ring systems subtend an inter-planar angle of 42.72 (5)°. In the crystal, mol­ecules are linked by N–H⋯N_nitrile_ and N–H⋯O=C hydrogen bonds, forming chains parallel to the *b* axis.

## Abstract 2

In the title compound 1-thio­phen-2-yl­methyl­ene­amino­pyrimidine derivative, the pyrimidine and thienyl rings are inclined to one another by 42.72 (5)°. In the crystal, mol­ecules are linked by N–H⋯N_nitrile_ and N–H⋯O=C hydrogen bonds, forming chains parallel to the *b* axis.

## Chemical context   

Pyrimidines are well known for their biological activities as anti­metabolic agents and have attracted much attention from the standpoint of pharmaceutical chemistry. Many drugs, such as 5-fluoro­uracil, containing a pyrimidine moiety have been developed and used as anti­cancer agents. It is difficult to find a general method for the introduction of specific substituents into the pyrimidine nucleus directly, and thus many synthetic methods have been developed for the construction of pyrimidine rings bearing potential functional groups (Elgemeie & Sood, 2001 ▸). As part of our program directed toward the preparation of potential anti­metabolites (Elgemeie & Hussain, 1994 ▸), we have recently reported various successful approaches for the syntheses of purine and pyrimidine analogues (Elgemeie, 2003 ▸; Elgemeie *et al.*, 2009 ▸). Derivatives of these ring systems are inter­esting as anti­metabolic agents in biochemical reactions (Scala *et al.*, 1997 ▸).
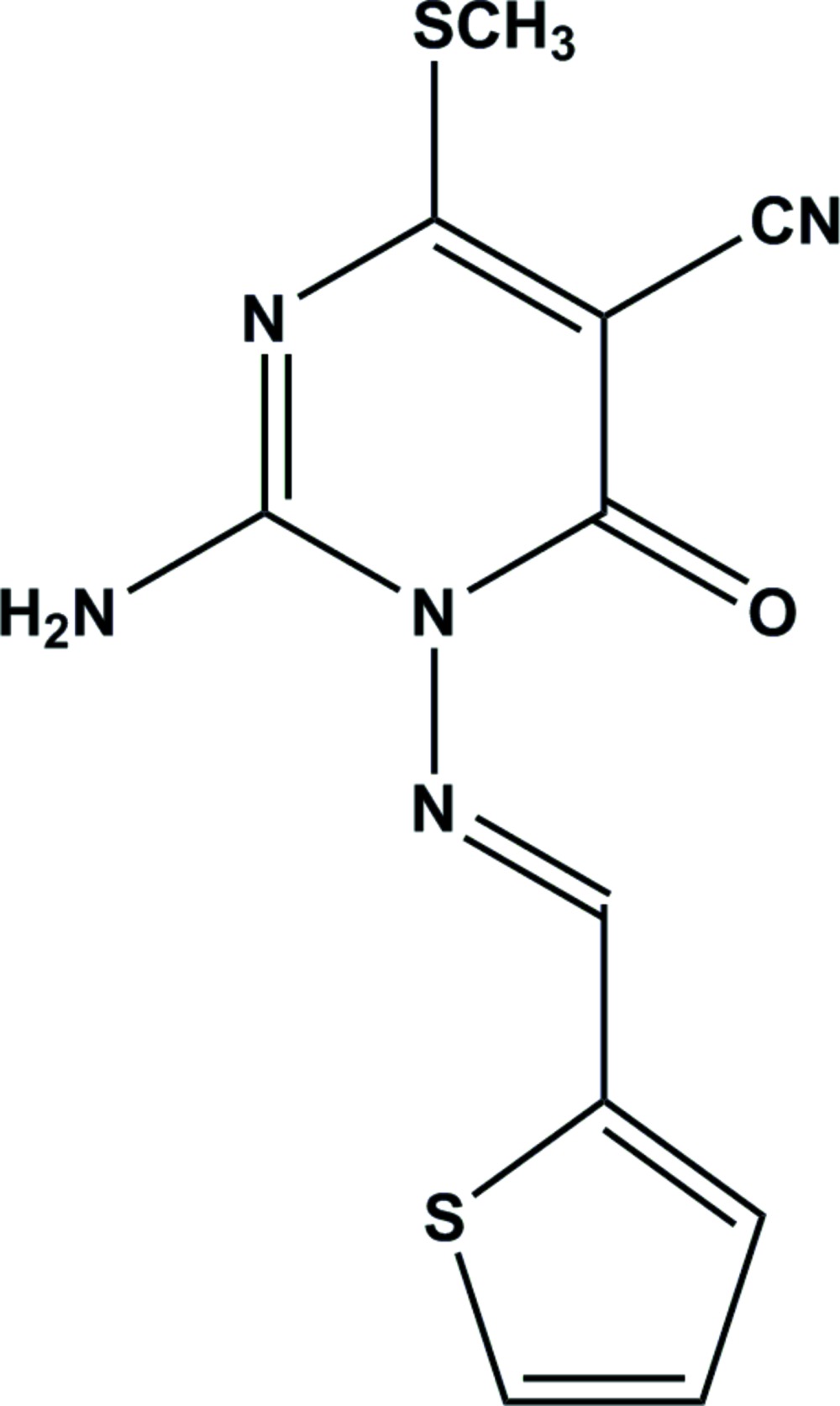


We report herein on the synthesis and crystal structure of a new 1-thio­phen-2-yl­methyl­ene­amino­pyrimidine derivative, obtained by reaction of dimethyl *N*-cyano­dithio­imino­carbonate with 1-cyano­acetyl-4-thio­phene­methyl­idene semicarbazide in dioxane containing KOH at room temperature. To the best of our knowledge, this is the first example of this approach to be reported for *N*-substituted amino­pyrimidine derivatives. The X-ray structure determination was undertaken to establish the nature of the product unambiguously.

## Structural commentary   

**Figure 1:**
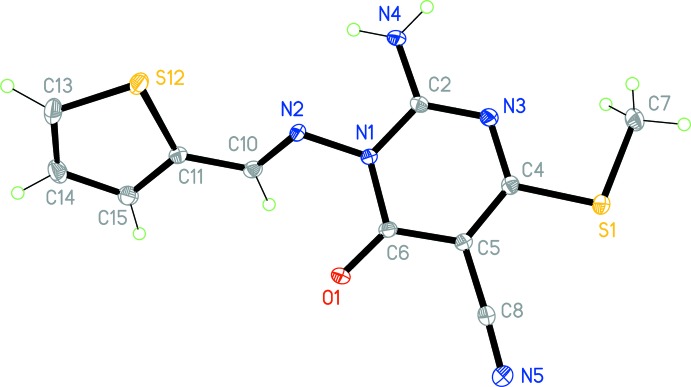
The mol­ecule structure of the title compound, with atom labelling. Displacement ellipsoids are drawn at the 50% probability level. The intra­molecular hydrogen bond, H042⋯N1, is not shown.

The mol­ecular structure of the title compound is shown in Fig. 1 ▸. The *E* conformation across the double bond N2=C10 is confirmed, with a bond length of 1.2879 (14) Å. Both ring systems are, as expected, planar (r.m.s. deviations are 0.017 Å for the pyrimidine and 0.001 Å for the thienyl ring). Atom N2 lies 0.189 (2) Å out of the pyrimidine plane; all other immediate substituent atoms lie effectively in the ring plane. Carbon atom C7 of the thio­methyl group is rotated slightly out of the ring plane, with torsion angle N3—C4—S1—C7 being −6.30 (10)°. The inter-planar angle between the rings is 42.72 (5)°; the relative orientation is influenced by the torsion angles C6—N1—N2—C10 = −51.78 (13), N1—N2—C10—C11 = 174.68 (9) and N2—C10—C11—S12 = 5.22 (15)°. The NH_2_ group is planar; the nitro­gen atom lies only 0.048 (9) Å out of the plane of its substituents. The intra­molecular contact H041⋯N2 = 2.22 (2) Å may be construed as a hydrogen bond, although the angle at the H atom is necessarily narrow at 108.4 (14) ° (Table 1 ▸).

## Supra­molecular features   

**Figure 2:**
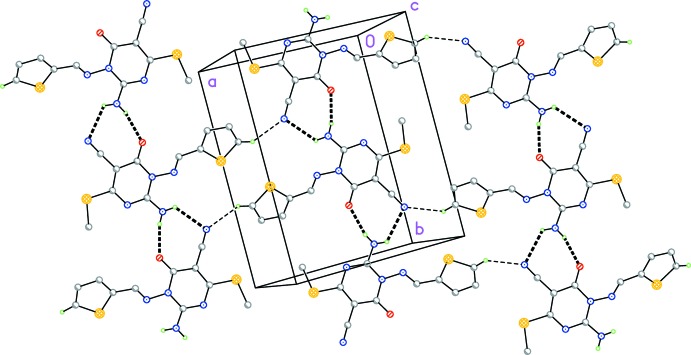
Crystal packing diagram of the title compound, viewed perpendicular to (102). Classical hydrogen bonds are drawn as thick dashed lines and ‘weak’ hydrogen bonds as thin dashed lines (see Table 1 ▸).

In the crystal, mol­ecules are connected into chains parallel to the *b* axis by the two classical hydrogen bonds, H041⋯N5 2.51 (2) Å and H042⋯O1 2.14 (2) Å (Table 1 ▸ and Fig. 2 ▸), both involving the 2_1_ screw operator −*x* + 1, *y* − 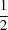, −*z* + 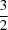. The longer of these two contacts forms part of a three-center system with the intra­molecular contact H041⋯N2 (Table 1 ▸). The ‘weak’ hydrogen bond H13⋯N5 of 2.49 Å, formed *via* the *c* glide operator *x* + 1, −*y* + 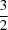, *z* − 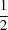, connects the chains to form layers parallel to (102) (Table 1 ▸ and Fig. 2 ▸).

## Database survey   

A search of the Cambridge Structural Database (Version 5.36, 2014; Groom & Allen, 2014 ▸) revealed 42 hits for pyrimidines with the amino and C=O functions located as for the title compound.

## Synthesis and crystallization   

Dimethyl *N*-cyano­dithio­imino­carbonate (0.01 mol) was added to a stirred solution of 1-cyano­acetyl-4-thio­phene­methyl­idenesemicarbazide (0.01 mol) in dry dioxane (50 ml), containing potassium hydroxide (0.01 mol), at room temperature. The solution was stirred overnight at room temperature, after which a colourless solid product was collected by filtration and crystallized from ethanol (m.p. 541–542 K), giving colourless block-like crystals.

## Refinement   

Crystal data, data collection and structure refinement details are summarized in Table 2 ▸. The NH hydrogen atoms were located in a difference Fourier map and freely refined. The C-bound H atoms were included in calculated positions and treated as riding atoms: C—H = 0.95–0.98 Å with *U*_iso_(H) = 1.5*U*_eq_(C) for methyl H atoms and 1.2*U*_eq_(C) for other H atoms. The methyl group was refined as an idealized rigid group, allowed to rotate but not to tip.

## Supplementary Material

Crystal structure: contains datablock(s) I, global. DOI: 10.1107/S205698901501885X/su5217sup1.cif

Structure factors: contains datablock(s) I. DOI: 10.1107/S205698901501885X/su5217Isup2.hkl

CCDC reference: 1430030

Additional supporting information:  crystallographic information; 3D view; checkCIF report

## Figures and Tables

**Table 1:** Hydrogen-bond geometry (, )

*D*H*A*	*D*H	H*A*	*D* *A*	*D*H*A*
N4H041N2	0.827(18)	2.224(17)	2.6062(13)	108.4(14)
N4H041N5^i^	0.827(18)	2.513(17)	3.0555(14)	124.2(15)
N4H042O1^i^	0.823(17)	2.144(17)	2.9414(12)	163.1(15)
C13H13N5^ii^	0.95	2.49	3.2722(15)	139
C10H10O1^iii^	0.95	2.35	3.2124(13)	150

Symmetry codes: (i) 
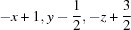
; (ii) 
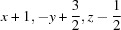
; (iii) 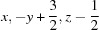.

**Table 2:** Experimental details

Crystal data	
Chemical formula	C_11_H_9_N_5_OS_2_
*M*_r_	291.35
Crystal system, space group	Monoclinic, *P*2_1_/*c*
Temperature (K)	100
*a*, *b*, *c* ()	11.4650(4), 14.7715(4), 7.5924(3)
()	96.397(3)
*V* (^3^)	1277.81(8)
*Z*	4
Radiation type	Mo *K*
(mm^1^)	0.42
Crystal size (mm)	0.40 0.40 0.12

Data collection	
Diffractometer	Oxford Diffraction Xcalibur, Eos
Absorption correction	Multi-scan (*CrysAlis PRO*; Agilent, 2013 ▸)
*T*_min_, *T*_max_	0.949, 1.000
No. of measured, independent and observed [*I* > 2(*I*)] reflections	40035, 3838, 3400
*R*_int_	0.035
(sin /)_max_ (^1^)	0.719

Refinement	
*R*[*F* ^2^ > 2(*F* ^2^)], *wR*(*F* ^2^), *S*	0.030, 0.078, 1.05
No. of reflections	3838
No. of parameters	181
H-atom treatment	H atoms treated by a mixture of independent and constrained refinement
_max_, _min_ (e ^3^)	0.45, 0.28

Computer programs: *CrysAlis PRO* (Agilent, 2013 ▸), *SHELXS97*, *SHELXL97* and *XP* in *SHELXTL* (Sheldrick, 2008 ▸).

## supplementary crystallographic information

### Crystal data

**Table:**

C_11_H_9_N_5_OS_2_	*F*(000) = 600
*M**_r_* = 291.35	*D*_x_ = 1.514 Mg m^−^^3^
Monoclinic, *P*2_1_/*c*	Mo *K*α radiation, λ = 0.71073 Å
Hall symbol: -P 2yc	Cell parameters from 11992 reflections
*a* = 11.4650 (4) Å	θ = 2.8–30.7°
*b* = 14.7715 (4) Å	µ = 0.42 mm^−^^1^
*c* = 7.5924 (3) Å	*T* = 100 K
β = 96.397 (3)°	Block, colourless
*V* = 1277.81 (8) Å^3^	0.40 × 0.40 × 0.12 mm
*Z* = 4	

### Data collection

**Table:**

Oxford Diffraction Xcalibur, Eos diffractometer	3838 independent reflections
Radiation source: Enhance (Mo) X-ray Source	3400 reflections with *I* > 2σ(*I*)
Graphite monochromator	*R*_int_ = 0.035
Detector resolution: 16.1419 pixels mm^-1^	θ_max_ = 30.7°, θ_min_ = 2.3°
ω–scan	*h* = −16→16
Absorption correction: multi-scan (*CrysAlis PRO*; Agilent, 2013)	*k* = −21→20
*T*_min_ = 0.949, *T*_max_ = 1.000	*l* = −10→10
40035 measured reflections	

### Refinement

**Table:**

Refinement on *F*^2^	Primary atom site location: structure-invariant direct methods
Least-squares matrix: full	Secondary atom site location: difference Fourier map
*R*[*F*^2^ > 2σ(*F*^2^)] = 0.030	Hydrogen site location: inferred from neighbouring sites
*wR*(*F*^2^) = 0.078	H atoms treated by a mixture of independent and constrained refinement
*S* = 1.05	*w* = 1/[σ^2^(*F*_o_^2^) + (0.0353*P*)^2^ + 0.5986*P*] where *P* = (*F*_o_^2^ + 2*F*_c_^2^)/3
3838 reflections	(Δ/σ)_max_ = 0.001
181 parameters	Δρ_max_ = 0.45 e Å^−^^3^
0 restraints	Δρ_min_ = −0.28 e Å^−^^3^

### Special details

**Table:**

Geometry. All e.s.d.'s (except the e.s.d. in the dihedral angle between two l.s. planes) are estimated using the full covariance matrix. The cell e.s.d.'s are taken into account individually in the estimation of e.s.d.'s in distances, angles and torsion angles; correlations between e.s.d.'s in cell parameters are only used when they are defined by crystal symmetry. An approximate (isotropic) treatment of cell e.s.d.'s is used for estimating e.s.d.'s involving l.s. planes.
Refinement. Refinement of *F*^2^ against ALL reflections. The weighted *R*-factor *wR* and goodness of fit *S* are based on *F*^2^, conventional *R*-factors *R* are based on *F*, with *F* set to zero for negative *F*^2^. The threshold expression of *F*^2^ > σ(*F*^2^) is used only for calculating *R*-factors(gt) *etc*. and is not relevant to the choice of reflections for refinement. *R*-factors based on *F*^2^ are statistically about twice as large as those based on *F*, and *R*- factors based on ALL data will be even larger.NH H atoms were refined freely. The methyl was refined as an idealized rigid group allowed to rotate but not tip (AFIX 137). Other H atoms were included using a riding model starting from calculated positions.

### Fractional atomic coordinates and isotropic or equivalent isotropic displacement parameters (Å^2^)

**Table:**

	*x*	*y*	*z*	*U*_iso_*/*U*_eq_	
S1	0.18882 (2)	0.570453 (19)	0.93852 (4)	0.01607 (8)	
N1	0.53629 (8)	0.58971 (6)	0.73366 (12)	0.01132 (17)	
C2	0.48455 (9)	0.50790 (7)	0.76239 (14)	0.01123 (19)	
N3	0.37941 (8)	0.50061 (6)	0.82064 (12)	0.01267 (18)	
C4	0.32601 (9)	0.57730 (7)	0.86044 (14)	0.01173 (19)	
C5	0.37518 (9)	0.66299 (7)	0.84526 (14)	0.0122 (2)	
C6	0.48602 (9)	0.67272 (7)	0.77860 (14)	0.01108 (19)	
C7	0.15734 (11)	0.45152 (8)	0.91405 (16)	0.0186 (2)	
H7A	0.1624	0.4334	0.7910	0.028*	
H7B	0.0781	0.4395	0.9450	0.028*	
H7C	0.2144	0.4169	0.9929	0.028*	
C8	0.31807 (10)	0.74448 (7)	0.88764 (15)	0.0147 (2)	
O1	0.53764 (7)	0.74475 (5)	0.75918 (11)	0.01462 (16)	
N2	0.65393 (8)	0.58528 (6)	0.69401 (13)	0.01224 (18)	
N4	0.54197 (9)	0.43358 (6)	0.72510 (14)	0.01410 (18)	
H041	0.6097 (16)	0.4387 (12)	0.699 (2)	0.027 (4)*	
H042	0.5115 (14)	0.3849 (11)	0.746 (2)	0.020 (4)*	
N5	0.27567 (9)	0.81204 (7)	0.92072 (15)	0.0213 (2)	
C10	0.67911 (9)	0.64245 (7)	0.57588 (14)	0.0123 (2)	
H10	0.6191	0.6792	0.5165	0.015*	
C11	0.79798 (9)	0.65073 (7)	0.53371 (14)	0.0124 (2)	
S12	0.91097 (2)	0.58149 (2)	0.62072 (4)	0.01827 (8)	
C13	1.01057 (10)	0.64133 (9)	0.51488 (16)	0.0190 (2)	
H13	1.0919	0.6273	0.5232	0.023*	
C14	0.96003 (11)	0.71124 (8)	0.41744 (16)	0.0189 (2)	
H14	1.0022	0.7516	0.3505	0.023*	
C15	0.83751 (10)	0.71713 (8)	0.42687 (15)	0.0157 (2)	
H15	0.7883	0.7616	0.3666	0.019*	

### Atomic displacement parameters (Å^2^)

**Table:**

	*U*^11^	*U*^22^	*U*^33^	*U*^12^	*U*^13^	*U*^23^
S1	0.01141 (13)	0.01430 (13)	0.02338 (15)	−0.00065 (9)	0.00585 (10)	−0.00028 (10)
N1	0.0097 (4)	0.0088 (4)	0.0158 (4)	0.0005 (3)	0.0028 (3)	−0.0004 (3)
C2	0.0122 (5)	0.0093 (4)	0.0120 (5)	−0.0008 (4)	0.0002 (4)	−0.0001 (3)
N3	0.0116 (4)	0.0110 (4)	0.0154 (4)	−0.0006 (3)	0.0014 (3)	−0.0001 (3)
C4	0.0099 (4)	0.0130 (5)	0.0121 (5)	−0.0001 (4)	0.0001 (4)	0.0006 (4)
C5	0.0110 (5)	0.0105 (4)	0.0153 (5)	0.0007 (4)	0.0024 (4)	−0.0004 (4)
C6	0.0107 (5)	0.0094 (4)	0.0130 (5)	0.0010 (3)	0.0007 (4)	−0.0008 (4)
C7	0.0168 (5)	0.0164 (5)	0.0235 (6)	−0.0054 (4)	0.0058 (4)	−0.0012 (4)
C8	0.0127 (5)	0.0136 (5)	0.0185 (5)	−0.0014 (4)	0.0045 (4)	0.0010 (4)
O1	0.0142 (4)	0.0086 (3)	0.0217 (4)	−0.0011 (3)	0.0044 (3)	−0.0012 (3)
N2	0.0090 (4)	0.0109 (4)	0.0173 (4)	0.0003 (3)	0.0035 (3)	−0.0014 (3)
N4	0.0134 (4)	0.0079 (4)	0.0214 (5)	−0.0002 (3)	0.0036 (4)	−0.0005 (3)
N5	0.0189 (5)	0.0158 (5)	0.0308 (6)	0.0014 (4)	0.0104 (4)	−0.0002 (4)
C10	0.0123 (5)	0.0115 (4)	0.0130 (5)	0.0009 (4)	0.0008 (4)	−0.0022 (4)
C11	0.0124 (5)	0.0111 (4)	0.0139 (5)	0.0009 (4)	0.0023 (4)	−0.0014 (4)
S12	0.01309 (14)	0.01868 (14)	0.02389 (16)	0.00414 (10)	0.00586 (11)	0.00657 (11)
C13	0.0128 (5)	0.0241 (6)	0.0210 (6)	−0.0025 (4)	0.0058 (4)	−0.0022 (5)
C14	0.0202 (6)	0.0181 (5)	0.0199 (5)	−0.0053 (4)	0.0084 (4)	−0.0004 (4)
C15	0.0181 (5)	0.0140 (5)	0.0158 (5)	0.0001 (4)	0.0048 (4)	0.0000 (4)

### Geometric parameters (Å, º)

**Table:**

S1—C4	1.7446 (11)	C11—C15	1.3811 (15)
S1—C7	1.7990 (12)	C11—S12	1.7234 (11)
N1—C2	1.3744 (13)	S12—C13	1.7133 (12)
N1—C6	1.4127 (13)	C13—C14	1.3617 (18)
N1—N2	1.4158 (12)	C14—C15	1.4172 (16)
C2—N4	1.3268 (13)	C7—H7A	0.9800
C2—N3	1.3337 (14)	C7—H7B	0.9800
N3—C4	1.3382 (13)	C7—H7C	0.9800
C4—C5	1.3956 (14)	N4—H041	0.827 (18)
C5—C8	1.4242 (15)	N4—H042	0.823 (17)
C5—C6	1.4265 (14)	C10—H10	0.9500
C6—O1	1.2343 (13)	C13—H13	0.9500
C8—N5	1.1500 (15)	C14—H14	0.9500
N2—C10	1.2879 (14)	C15—H15	0.9500
C10—C11	1.4395 (14)		
			
C4—S1—C7	101.52 (5)	C10—C11—S12	123.65 (8)
C2—N1—C6	122.00 (9)	C13—S12—C11	91.48 (6)
C2—N1—N2	115.55 (8)	C14—C13—S12	112.37 (9)
C6—N1—N2	121.03 (8)	C13—C14—C15	112.54 (10)
N4—C2—N3	119.49 (10)	C11—C15—C14	112.22 (10)
N4—C2—N1	117.42 (10)	S1—C7—H7A	109.5
N3—C2—N1	123.07 (9)	S1—C7—H7B	109.5
C2—N3—C4	117.37 (9)	H7A—C7—H7B	109.5
N3—C4—C5	123.36 (10)	S1—C7—H7C	109.5
N3—C4—S1	118.70 (8)	H7A—C7—H7C	109.5
C5—C4—S1	117.93 (8)	H7B—C7—H7C	109.5
C4—C5—C8	123.21 (10)	C2—N4—H041	118.5 (12)
C4—C5—C6	120.31 (9)	C2—N4—H042	116.8 (11)
C8—C5—C6	116.44 (9)	H041—N4—H042	123.9 (16)
O1—C6—N1	120.35 (9)	N2—C10—H10	119.9
O1—C6—C5	125.96 (10)	C11—C10—H10	119.9
N1—C6—C5	113.69 (9)	C14—C13—H13	123.8
N5—C8—C5	177.44 (12)	S12—C13—H13	123.8
C10—N2—N1	114.23 (9)	C13—C14—H14	123.7
N2—C10—C11	120.12 (10)	C15—C14—H14	123.7
C15—C11—C10	124.88 (10)	C11—C15—H15	123.9
C15—C11—S12	111.39 (8)	C14—C15—H15	123.9
			
C6—N1—C2—N4	−176.23 (10)	N2—N1—C6—C5	−169.29 (9)
N2—N1—C2—N4	−9.70 (14)	C4—C5—C6—O1	179.88 (11)
C6—N1—C2—N3	5.62 (16)	C8—C5—C6—O1	2.07 (17)
N2—N1—C2—N3	172.15 (10)	C4—C5—C6—N1	−0.23 (15)
N4—C2—N3—C4	178.49 (10)	C8—C5—C6—N1	−178.04 (9)
N1—C2—N3—C4	−3.40 (16)	C2—N1—N2—C10	141.55 (10)
C2—N3—C4—C5	−0.54 (16)	C6—N1—N2—C10	−51.78 (13)
C2—N3—C4—S1	−179.55 (8)	N1—N2—C10—C11	174.68 (9)
C7—S1—C4—N3	−6.30 (10)	N2—C10—C11—C15	−171.31 (11)
C7—S1—C4—C5	174.63 (9)	N2—C10—C11—S12	5.22 (15)
N3—C4—C5—C8	179.96 (11)	C15—C11—S12—C13	0.11 (9)
S1—C4—C5—C8	−1.01 (15)	C10—C11—S12—C13	−176.84 (10)
N3—C4—C5—C6	2.31 (17)	C11—S12—C13—C14	0.08 (10)
S1—C4—C5—C6	−178.67 (8)	S12—C13—C14—C15	−0.25 (14)
C2—N1—C6—O1	176.40 (10)	C10—C11—C15—C14	176.64 (10)
N2—N1—C6—O1	10.60 (15)	S12—C11—C15—C14	−0.26 (13)
C2—N1—C6—C5	−3.50 (15)	C13—C14—C15—C11	0.33 (15)

### Hydrogen-bond geometry (Å, º)

**Table:**

*D*—H···*A*	*D*—H	H···*A*	*D*···*A*	*D*—H···*A*
N4—H041···N2	0.827 (18)	2.224 (17)	2.6062 (13)	108.4 (14)
N4—H041···N5^i^	0.827 (18)	2.513 (17)	3.0555 (14)	124.2 (15)
N4—H042···O1^i^	0.823 (17)	2.144 (17)	2.9414 (12)	163.1 (15)
C13—H13···N5^ii^	0.95	2.49	3.2722 (15)	139
C10—H10···O1^iii^	0.95	2.35	3.2124 (13)	150

Symmetry codes: (i) −*x*+1, *y*−1/2, −*z*+3/2; (ii) *x*+1, −*y*+3/2, *z*−1/2; (iii) *x*, −*y*+3/2, *z*−1/2.
